# Healthcare professional perspectives on hereditary breast cancer risk assessment prior to gender-affirming mastectomy

**DOI:** 10.1007/s10549-026-08038-9

**Published:** 2026-07-29

**Authors:** Kimberly Zayhowski, Kai Blumen, Kathleen F. Mittendorf, Rebekah Pratt, Tala Berro, Carl Streed, Ian M. MacFarlane

**Affiliations:** 1 Medical Sciences and Education, Boston University Chobanian and Avedisian School of Medicine, 72 East Concord Street, Boston, MA 02118 USA; 2 Department of Genetics, Cell Biology and Development, University of Minnesota, Minneapolis, MN USA; 3 Master’s Program in Genetic Counseling, Boston University Chobanian and Avedisian School of Medicine, Boston, MA USA; 4 Lavender Spectrum Health, Vancouver, WA USA; 5 Department of Family Medicine and Community Health, University of Minnesota, Minneapolis, USA; 6 Department of Genetics, Kaiser Permanente, Oakland, CA USA; 7 Section of General Internal Medicine, Boston University Chobanian and Avedisian School of Medicine, Boston, MA USA; 8 GenderCare Center, Boston Medical Center, Boston, MA USA

**Keywords:** Transgender, Mastectomy, Genetic counseling, Gender-affirming care, Breast cancer, Reflexive thematic analysis

## Abstract

**Purpose:**

Transgender and gender diverse (TGD) individuals undergoing gender-affirming mastectomy (GAM) face unique challenges related to breast cancer risk assessment and screening. This study aimed to identify factors that influence healthcare professionals’ (HCPs) integration of cancer risk evaluation, including genetic assessment, within the context of GAM and pre-surgical planning.

**Methods:**

We conducted semi-structured qualitative interviews with 20 healthcare professionals - including six primary care providers, five cancer genetic counselors, six oncologists, and three plastic surgeons - experienced in caring for TGD patients considering or undergoing GAM. Using a social constructivist framework, we applied reflexive thematic analysis to explore HCP perspectives and generate key themes.

**Results:**

We conceptualized five key themes: (1) HCPs perceive no clear ownership for care integration of breast cancer risk assessment; (2) conflicting guidelines force HCPs to rely on their own judgment, resulting in inconsistent practice; (3) care pathways are driven by individual HCP champions rather than standardized protocols; (4) GAM frequently occurs at young ages prior to age of population cancer risk screening initiation, complicating risk discussions; and (5) structural failures in care delivery compromise patient safety.

**Conclusion:**

Improving cancer risk assessment for TGD patients undergoing GAM demands clear institutional accountability, harmonized evidence-based guidelines, standardized care pathways, and genetic counseling embedded into multidisciplinary gender-affirming care teams. These coordinated structural efforts are critical to integrate equitable, personalized, and patient-centered cancer prevention with gender-affirming surgical care.

**Supplementary Information:**

The online version contains supplementary material available at 10.1007/s10549-026-08038-9.

## Introduction

Transgender and gender diverse (TGD) individuals experience significant healthcare disparities across the oncology spectrum of care [1, 2]. Systemic barriers, past harmful healthcare experiences, and sociopolitical climate, among others, combine to influence these disparities [1–5]. There are disparities in breast cancer preventive screening adherence in TGD people assigned female at birth compared to cisgender women (2–50% vs. 65–78%), and TGD people and their healthcare professionals (HCPs) are often unaware of TGD-tailored screening guidelines [5–8]. Given screening’s critical role in early diagnosis, it is unsurprising that breast cancer diagnosis in TGD persons often occurs at later stages [9–11].

Precision oncology aims to tailor cancer prevention strategies to an individual patient’s unique cancer risks. TGD people who elect gender-affirming medical care have surgeries and/or exogenous hormone use that can alter cancer risk profiles [12–15]. However, a dearth of available data leaves care gaps in precision medicine strategies related to these altered risk profiles, including a lack of consistent guidelines that are infrequently implemented in practice [16].

Gender-affirming mastectomy (GAM, colloquially “top surgery”) represents one such risk profile-altering intervention. GAM removes some – but not all – breast tissue, leaving enough remaining tissue to construct a masculine chest contour [17–20]. GAM contrasts with classic risk-reducing mastectomy (RRM), which is frequently performed prophylactically on cancer-unaffected people assigned female at birth at high genetic risk [21–23] and which removes all breast tissue between the epithelium and muscle wall. As a result, individuals receiving GAM have a reduced risk compared to cisgender women, but a higher risk than cisgender men [15, 24], with the highest estimates suggesting a breast cancer incidence 60 times that of cisgender men (about 0.4 times that of cisgender women) [15].

GAM is often undertaken at relatively young ages, with studies reporting a range of median ages (21.7–27 years) [25–27]. Young patients and their HCPs may not be focusing on breast cancer risk. Yet, 10–20% of breast cancers are caused by hereditary risk factors, and the most common cancer germline cancer risk variants are associated with breast cancer [28, 29]. Given the differences between RRM and GAM, a thorough pre-surgical risk assessment could inform surgery technique, providing a key avenue to close precision oncology care gaps for TGD people [24, 30–33]. While some professional organizations and transgender health guidelines recommend pre-surgical risk evaluation [20, 30, 34], implementation strategies are not well defined, limiting reach and effectiveness of cancer prevention for TGD individuals.

Recent research foregrounds the patient perspective, revealing substantial patient uncertainty about post-GAM cancer screening, limited HCP knowledge of risks following GAM, and the impact of feminized breast health spaces on patient comfort and care engagement [33]. Further, an early pilot study suggests that when provided education about their risk, about half of participants at elevated risk opt for an RRM rather than traditional GAM [32].

Despite the U.S. Preventive Services Task Force (USPSTF) recommendations for breast cancer risk assessment in primary care since 2013, there are substantial gaps in appropriate risk assessment and referral [35, 36]. HCPs report discomfort and limited knowledge regarding genetic evaluation and hereditary cancer, as well as TGD specific breast cancer screening [37–39]. Combined with conflicting guidelines for TGD-specific care [16], this discomfort may exacerbate care gaps in precision medicine in ways that specifically impact TGD people. To explicate these gaps, we conducted qualitative interviews with HCPs to identify factors influencing breast cancer risk assessment and follow-up care, ultimately aiming to enhance shared decision-making, improve clinical outcomes, and advance precision breast oncology for TGD people.

## Methodology

A detailed account of our methodology and its rationale resides in Supplemental Materials I. Briefly, we aimed to identify HCP-level factors that influence breast cancer risk assessment, screening uptake, and the perceived utility/acceptability of cancer genetic counseling for TGD patients. We conducted semi-structured interviews with HCPs, including primary care providers, cancer genetic counselors, breast oncologists and surgical oncologists, and plastic surgeons providing care to TGD individuals considering or undergoing top surgery. Our research team included experts in genetic counseling, cancer care, research methodology, and transgender health, a large portion of whom are LGBTQ+ community members, including several who are TGD. This study was approved by the Boston University Institutional Review Board (IRB Protocol H-44213) in October 2023. Its companion study on TGD patient perspectives was published previously [33].

### Participant recruitment

We recruited nationally through professional networks, organizational listservs, and cancer genetics and transgender health platforms, followed by snowball sampling. Interested HCPs completed a brief demographic survey (see Supplemental Materials II) via Qualtrics. We used purposeful sampling to ensure diversity in professional role, geographic region, years of experience, gender, and race/ethnicity. We intentionally oversampled professionals with higher prior experience working with TGD patients and/or managing patients with familial cancer risk. To be eligible for the study, participants had to (1) be ≥ 18 years old, (2) speak English, (3) be currently practicing in genetic counseling, primary care, surgical or medical oncology, or plastic surgery in the United States, and (4) have direct experience caring for TGD patients considering or undergoing top surgery. We continued recruitment until we determined we had achieved sufficient informational power based on interim analysis of interview depth and quality [40].

### Data generation

The interview guide (see Supplemental Materials III) gathered in-depth perspectives from HCPs on several topics relevant to TGD breast cancer risk assessment. We included questions about current practices and awareness, incorporation of breast cancer and genetic risk evaluation, knowledge about RRM and GAM differences, and patient communication and informed decision-making. Pilot interviews with two cancer genetic counselors (not included in analysis) were used to guide minor interview guide adjustments for clarity. Secure video interviews were conducted by KB between June and October 2024. KB and KZ conducted reflexive journaling and debriefed following each interview, facilitating ongoing data familiarity and preliminary analyses. Participants received a $50 Amazon gift card.

Interviews were audio-recorded and transcribed verbatim. We used NVivo software (version 14.23.4) [41] to organize and code transcripts. Two team members (KB and KZ) coded the data inductively and collaborated with two other members (KFM and IMM) to regularly review the data and preliminary themes. Reflexive thematic analysis was performed to conceptualize key themes [42–45]. Throughout our analysis, we embraced a social constructivist perspective, acknowledging that we co-constructed the themes in partnership with our participants and that factors such as lived experiences, workplace contexts, and power dynamics influenced meanings derived [46]. Given the exploratory nature of our research and the limited prior studies on HCP perspectives in this area, we intentionally chose not to impose a predetermined theoretical framework during analysis. The team iterated theme development, selected illustrative quotes, and collectively drafted and revised the manuscript.

## Results

We analyzed interviews with 20 HCPs: six primary care providers (PCP), five genetic counselors (GC), five surgical oncologists (SO), three plastic surgeons (PS), and one medical oncologist (MO) (see Table 1 for demographics). The mean interview time was 42 min (range 22–92 min). We constructed five key themes (see Table 2 for additional illustrative quotes).

**Table 1:** Participant demographic information

Item	Response	Participants (*N* = 20)*
Race/ethnicity *Select all that app* *ly*	White	17
	Asian	2
	Black, African American, or African	2
	Middle Eastern	1
Gender *Select all that apply*	Woman	10
	Man	7
	Genderqueer	3
	Non-binary	2
	Agender	1
	Not listed *(please specify)*	
	Transgender male	1
Specialty	Primary care provider	6
	Genetic counselor	5
	Surgical oncologist	5
	Plastic surgeon	3
	Medical oncologist	1
State of practice	Massachusetts	5
	Pennsylvania	4
	California	2
	Illinois	2
	Minnesota	2
	Connecticut	1
	Michigan	1
	New Jersey	1
	Tennessee	1
	Wisconsin	1
Years of practice	Board eligible	2
	1–4	8
	5–9	4
	10–14	4
	15+	2
Type of clinical practice	Academic medical center	17
	Community practice	2
	Not listed (*please specify*)	
	College health	1
Types of health insurance common in practice *Select all that apply*	Public health insurance	19
	Private health insurance	16
	No health insurance	5

*The total responses for several response options are greater than 20 due to the fact that several questions (race/ethnicity, gender) allowed for multiple selection. Row-level data were not included for participant privacy

## Theme I: HCPs perceive no clear ownership for care integration of breast cancer risk assessment

HCPs consistently described a lack of clear ownership regarding responsibility for conducting breast cancer risk assessments within GAM. This diffusion of responsibility led to missed opportunities for early identification of patients at risk, as no single provider or specialty felt fully accountable for integrating risk assessment into clinical workflows. Several participants highlighted deferment of screening and risk discussions to other specialists, leaving gaps in recommendations related to breast risk. For example, one genetic counselor expressed:

I sort of love the phrase, defer further cancer screening to their specialty care team, not me. (GC1)

While a primary care provider noted reliance on surgeons for screening decisions, a surgical oncologist deferred to primary care to initiate risk assessment while acknowledging a need for dedicated attention:

Anyone who’s high risk – the surgeon will hopefully do that screening and determine if someone needs total tissue removal versus sometimes, with more cosmetics, some tissue can be left behind. (PCP3)

Well, it’s thinking about it and incorporating real formal risk assessment into the encounters. And I think that probably often should begin in primary care, but it just requires attention to it and somebody to just think about that this is really an important thing to do before going down this pathway. (SO2)

## Theme II: Conflicting guidelines force HCPs to rely on their own judgment, resulting in inconsistent practice

Participants reported that conflicting institutional and national guidelines complicated efforts to adopt a standardized approach for genetic risk assessment and screening. In the absence of clear and consistent recommendations in the context of TGD care, HCPs relied heavily on personal judgment, creating variability in clinical practices and patient outcome disparities. A plastic surgeon summarized the situation:

I don’t think the guidelines are very clear. And so between the different societies and between even the American College of Radiology, and so that makes it a little bit confusing. I know that WPATH [World Professional Association for Transgender Health] has their own set of basically encouraged guidelines, but … it wasn’t set in stone in terms of specific ages or the recommendations that are there for cisgender individuals. And so it’s somewhat confusing. (PS3)

## Theme III: Care pathways are driven by individual HCP champions rather than standardized protocols

Rather than following standardized protocols, care pathways often hinged on individual HCPs advocating for genetic risk assessment from personal interest/expertise. These champions advanced genetic services locally, highlighting the need for formal guidelines to ensure equitable and systematic integration across care settings. One primary care provider emphasized the importance of local champions to drive change:

It’s really important that every healthcare location, clinic … has a champion for these issues because they can help be the person to go to as to ask questions, to help, to push for change. … And so empowering individual providers to remind them that you are the expert in this field, you do have the authority to go out and make this change. (PCP6)

A genetic counselor reiterated the impact of individual advocates in educating and inspiring others to incorporate risk assessment into their clinical practice:

[A genetic counselor] … made [an online module on transgender care], … I took a lot of that and a lot of information from presentations [genetic counselors] have done. … And then some of my own research and reading the ACR [American College of Radiology] guidelines and all of that together. And I give presentations now on this topic. … I am always met with, ‘We are so glad that you gave us this information because we did not know where to go for it. We did not know what to do.’ … And [then] they feel empowered to do things. (GC3)

## Theme IV: GAM frequently occurs at young ages prior to age of population cancer risk screening initiation, complicating risk discussions

Participants highlighted challenges discussing breast cancer risks for patients undergoing gender-affirming top surgery, as the timing of these procedures is often earlier than population breast cancer screening age. Pre-surgical planning at younger ages complicates shared decision-making and risk communication. A genetic counselor described conversations about the complexities of genetic testing in younger patients, especially when known familial pathogenic variants for hereditary cancer risk could influence surgical planning and earlier screening decisions:

We have talked to patients about, you could do a normal top surgery and then if you wanted to do something preventive later, but we’re typically not in favor of that. … We’d rather you not have multiple surgeries and potentially be at risk. (GC2)

A surgical oncologist further explained the difficulty in forecasting risk for younger patients:

Most patients in this situation of top surgery, … are fairly young for considering one’s cancer risk. Cancer risk becomes top of mind probably around 40 or so, and if you’re in your twenties and doing this, that’s not top of mind. And so how do we forecast or help look into the future? (SO4)

## Theme V: Structural failures in care delivery compromise patient safety

Several HCPs expressed concern that structural failures in care processes, including inadequate assessment of residual chest tissue and delays in examination, risk patient safety. One primary care provider recounted a tragic case illustrating these concerns:

There was not adequate assessment of the fact that he had residual chest tissue and there was a delay to an exam and a delay to recognizing that he had a lump – actually had a very significant one – and ultimately went on to be diagnosed with very advanced disease and died. … It has stayed with me over the years. You need to know that there’s chest tissue there. (PCP1)

**Table 2:** Illustrative quotes for themes

Theme	Quote
	*Participant quotes are arranged sequentially by provider type and participant number*
(1) HCPs perceive no clear ownership for care integration of breast cancer risk assessment	“In general in healthcare, everything’s in a silo. It would be really nice to have a comprehensive team of people who are involved with someone seeking surgery.” PCP2
	“It boils down to … providing good education. So from my end, I’m talking about patients with mastectomies, and I’m hoping that the surgical providers are doing this since they’re the ones doing the surgery, but hard to say. … The more information that you can provide, the better.” PCP6
	“I do worry that some patients will just not follow through. If I was really concerned about someone, if they had particularly high risk, I would probably push them a little bit more or make sure that I then circled back with whoever the referring provider was for them, their primary care … to just be like, ‘Hey, you might want to follow up with this person.’” PS1
(2) Conflicting guidelines force HCPs to rely on their own judgment, resulting in inconsistent practices	“Even when I have the patients who could meet criteria for testing, … I’m just very upfront. … ‘There’s still a lot of research going on. We don’t have a lot of data. Things are kind of up in the air, which is really unfortunate.’ So I do often tell them if we do happen to find that there is an increased risk that could affect surgical recommendations, we would have a more specialized conversation about that so that we could tailor it more to them and figure out what would be the best option for them based off of their journey.” GC4
	“WPATH [World Professional Association for Transgender Health] basically says follow national guidelines. I know to go to the UCSF [University of California San Francisco] trans excellence guideline, but I also know that what’s written there is basically expert opinion. … There’s been some interesting stuff in the American College of Radiology … [but] the radiologist lens on this is going to be a little different than the general population screening lens.” PCP1
	“I say we’re in an unknown space, whatever chest tissue is remaining, if there’s enough breast tissue, particularly that risk might be present, but we don’t know what the appropriate screening protocol looks like.” PCP4
	“Having more clear guidance would be helpful. … I go to the literature and try to look periodically so that I make sure I’m keeping up to date with things. … It would be nice if it was just a one-stop shop kind of thing.” PS1
(3) Care pathways are driven by individual HCP champions rather than standardized protocols	“I started more of a collaboration with our gender development program. They really pulled me in more because they were identifying patients that needed us, and then we helped to provide them with some more education on what family history features they could use to identify patients. So now we have a pretty consistent stream of patients that are being identified. We’re working to formalize that further.” GC2
	“Not everybody is sitting with [a knowledgeable genetic counselor] that they can just immediately refer people to. … A lot of PCPs would not have somebody clear to refer to around high risk for breast or chest cancer in anybody. Honestly, that’s already a clinical gap. And then to know that that referral partner was going to be aware of trans and gender diverse issues or even the basics.” PCP1
	“Part of the role of a person who practices medicine, surgery, plastic surgery, and gender affirmation surgery is you have to equip yourself with the knowledge required for giving the advice for the individual. Because [patients are] trusting you with your knowledge for the best advice possible. … There may not be very solid level one evidence for everything that you’re looking for, but part of your role as a practitioner is to collect the best evidence possible and use that as the basis for your advice gathering and advice giving to the patients.” PS2
(4) Gender affirming mastectomies frequently occur at young ages prior to age of population cancer risk screening initiation, complicating risk discussions	“We don’t typically use any calculators just for our patients because they’re typically not applicable to patients under 18. So we don’t even go there. Sometimes for our older patients we might use a Tyrer-Cusick or things like that if they’re for a hereditary breast cancer situation, but we typically haven’t used risk calculators.” GC2
	“We’re talking about top surgery in the twenties. When should we get them tested for the *BRCA*? What is the plan there? And so we haven’t done anything there yet with the kiddos, but we’re having these broader conversations.” GC3
	“A lot of patients that I see right now, they’re younger, usually they’re under 40 and they’re not really doing chest screenings at this point.” GC4
	“We have seen several times the example where it’s younger patients, so there’s no real obvious reason to do screening imaging before surgery and then finding incidental cancers when they undergo the top surgery.” SO2
	“Some [patients] do think about breast cancer as they get older, but younger people, we don’t think about cancer. 25 year olds aren’t being worried about cancer, at least the majority aren’t for my experience.” SO5
(5) Structural failures in care delivery compromise patient safety	“It really shocked me that in 2023, this was the first *BRCA* positive trans patient that they’d seen with prior top surgery and were like, ‘Oh, this is so novel.’ So I think that that was a big wake up call. … People aren’t educating themselves on this and are just not thinking about it until they’re confronted with it.” GC5
	“[I had] a patient who had had top surgery elsewhere in a different state several years ago, but then came in with a palpable mass in the chest wall, which ended up being breast cancer. And then I have had one or two who’ve had incidental cancers, which were really only found incidentally in the specimens having had top surgery.” SO2

GC = genetic counselor, PCP = primary care provider, PS = plastic surgeon, SO = surgical oncologist

## Discussion

This qualitative study with HCPs highlights key structural and systemic gaps that hinder effective integration of genetic risk assessment and breast cancer prevention in the GAM surgical pathway for TGD patients, including diffusion of responsibility, guideline fragmentation, reliance on individual champions, timing mismatch between GAM and standard screening ages, and failures in postoperative surveillance. Addressing these barriers requires urgent, coordinated changes at multiple levels of healthcare delivery to advance equity and precision oncology outcomes.

At the foundation, healthcare systems must establish clear institutional ownership of and accountability for breast cancer risk evaluation, especially within GAM pathways. Consistent with prior literature [47–51], we found diffusion of responsibility likely contributes to inconsistent practices and missed early risk identification, which may impact surgical choice and post-surgical screening interventions in GAM patients. Also consistent with past literature in cisgender patients, we found that implementation of standardized clinical workflows that assign explicit roles, particularly empowering primary care providers to initiate family history and risk assessment in accordance with USPSTF guidelines, with seamless genetic and surgical referrals, is critical [36, 37].

While there have been many studies evaluating interventions in this realm [38, 48, 52–54], only one pilot study has focused on the implications of risk assessment on GAM [32]. Including genetic risk assessment in the presurgical GAM pathway is critical to provide these frequently young patients with appropriate guidance impacting their surgical choice and to move beyond the reliance on individual clinical champions. Past work shows TGD patients report desire for such integration, indicating likely acceptability and adoption in this population [33]. Institutional commitment to genetics roles, whether via co-located genetic counselors, telehealth integration, or HCP cross-training, is essential to ensure equitable access and sustainability. Moreover, system-wide HCP education programs are needed to improve genetic literacy and normalize risk assessments as a routine component of primary care, reducing disparities caused by variable HCP knowledge and comfort [37, 38]. Lastly, risk assessment and cancer prevention programs, including screening, should be gender-inclusive, accounting for patient-reported gender dysphoria in feminized breast screening experiences and the importance of gender-inclusive care [5–7, 16, 33, 55–57].

Equally imperative is the development and widespread adoption of unified, evidence-based guidelines tailored to the unique cancer risk profiles of TGD patients, a process which requires ongoing detailed data ascertainment efforts on cancer risk and outcomes in this population. The misalignment between typical ages of gender-affirming surgery and standard cancer screening guidelines calls for innovative longitudinal risk models that account for such factors as hormone exposure, age at hormone initiation, history of gender-affirming oophorectomy, age at top surgery, and top surgery technique. Furthermore, existing quantitative risk tools such as the Tyrer-Cuzick and Gail models were developed and validated in cisgender women, and do not incorporate these variables, limiting their direct applicability for TGD people [58, 59]. Policies impacting data ascertainment practices – particularly those which restrict collection of gender identity and related gender-affirming care practices – impact the precision of models that will derive from these data [60–64]. National oncology, genetics, and transgender health organizations must collaborate to harmonize recommendations covering preoperative risk assessment, genetic testing indications, and postoperative surveillance that accommodate anatomical and hormonal differences, and initiate longitudinal data ascertainment efforts that account for TGD-specific care. Creating unified evidence-based guidelines and decision support frameworks that enable personalized, lifespan risk forecasting would reduce HCP uncertainty, standardize care, and facilitate precision detection and prevention. Like efforts in cisgender-centric care [65–71], ultimately providing patients and providers with dynamic, data-informed tools can facilitate shared decision-making that balances cancer prevention with gender affirmation, ensuring surgical choices and surveillance plans align with individual risk and needs.

Finally, standardizing postoperative surveillance protocols – accounting for individualized risk – to ensure evaluation of residual breast/chest tissue must become a quality metric. Systems should implement reminders, staff training, and performance monitoring to prevent delays in cancer detection and enhance patient safety. Insurance coverage for genetic counseling, risk assessment, and appropriate screening must be expanded and explicitly include gender-affirming care contexts, recognizing that legal sex may be associated with breast screening guidelines inappropriate to this population. Advocacy by professional organizations and patient communities can drive necessary reimbursement reforms and support resource allocation tailored to TGD care needs.

### Limitations and future directions

Key limitations affecting the transferability of findings include the small number of individuals in each represented specialty, geographic concentration of practice settings, and a predominance of participants from academic medical centers. Additionally, sampling methods may have increased the participation of providers more familiar with genetics through selective interest in study participation. Larger provider surveys and implementation science studies could build on this study to create systems-level interventions that account for provider knowledge and health system-level differences (e.g., academic vs. community practice needs).

## Conclusion

Addressing breast cancer risk assessment and prevention in TGD individuals requires coordinated structural initiatives that prioritize equity and precision. Establishing clear accountability within clinical workflows, developing comprehensive TGD-specific guidelines, and integrating genetic counseling into multidisciplinary care are foundational steps. Implementing standardized postoperative surveillance will improve clinical decision-making and patient safety. Advancing personalized risk modeling by re-establishing data ascertainment about TGD individuals in large cancer studies is critical for precision oncology. Supportive policies that enhance insurance coverage are needed to ensure access to these services. These actions are necessary to enhance cancer prevention, promote inclusive and response care, and transform precision oncology for TGD patients.

## Supplementary Information

Below is the link to the electronic supplementary material.

Supplementary Material 1

Supplementary Material 2

Supplementary Material 3

## Data Availability

Research data are not shared due to the remote risk of re-identifying a participant.

## Declarations

### Disclaimers

The manuscript is an original research article, whose findings have not been published elsewhere, are not submitted elsewhere for review, and have not been presented in full.
