# Supplementary material for: Healthcare professional perspectives on hereditary breast cancer risk assessment prior to gender-affirming mastectomy
Source: Breast Cancer Res Treat. 2026 Jul 29;218(2):22. doi: 10.1007/s10549-026-08038-9 (PMC13421360; doi:10.1007/s10549-026-08038-9)
Supplement: Supplementary file 1 — Supplementary Material 1 [file 10549_2026_8038_MOESM1_ESM.docx]

### **SUPPLEMENTAL MATERIALS I: METHODOLOGY**

####

#### **Study Design and Conceptual Framework**

We conducted qualitative interviews to identify healthcare provider (HCP)–level influences on breast cancer risk assessment, screening practices, and the use and perceived value of cancer genetic counseling when managing transgender and gender diverse (TGD) patients who are considering or undergoing top surgery (both traditional gender-affirming mastectomy [GAM, or “top surgery”] and risk-reducing mastectomy [RRM]). The goal was to produce practice‑focused insights that can inform clinician‑facing, evidence‑informed interventions to improve equitable cancer prevention and counseling within gender‑affirming care. Our research team combined expertise in cancer genetics, prevention, surgical and primary care, and qualitative methods, and included multiple LGBTQ+ members, including TGD investigators. One team member had personal experience with cancer genetic testing relevant to top‑surgery decision‑making.

We selected a qualitative approach to capture the complex nuances of HCP decision-making, workplace and structural contexts, and the interpersonal factors influencing care for TGD individuals – all dimensions not readily accessible through standardized instruments. We conducted semi-structured interviews to facilitate open-ended discussion, allowing participants to share candid perspectives, in-depth experiences, and beliefs related to breast cancer risk evaluation, genetic counseling and testing, and gender-affirming surgical options for TGD patients.

The study adopted a social constructivist paradigm^1^ to examine how clinicians and organizations jointly create meanings around cancer risk and surgical decision‑making in gender‑affirming contexts. Because our focus was exploratory and centered on professional sense‑making, we used inductive reflexive thematic analysis^2–5^ rather than applying a predetermined theoretical framework, allowing themes to be conceptualized from providers’ accounts and workplace practices.

We received approval from the Boston University Institutional Review Board (IRB Protocol H-44213) in October 2023. Its companion study, which reports findings from TGD patients’ perspectives on cancer risk assessment, genetic counseling, and surgical decision making, was published previously.^6^ The current manuscript presents complementary data from HCP interviews using a separate analytic sample.

#### **Participant Recruitment and Eligibility**

Healthcare professionals were recruited nationally (June–October 2024) via targeted outreach to specialty organizations, professional listservs, and clinician networks, with supplementary snowball sampling. Interested HCPs completed an online screening and demographic intake survey in Qualtrics (see Supplemental Materials II). Our purposive sampling prioritized breadth across clinician type (genetic counselors, primary care clinicians, breast oncologists/surgeons, plastic surgeons), geographic region, clinical experience, and demographic diversity. We oversampled clinicians with substantive experience caring for TGD patients or managing familial cancer risk to ensure detailed, practice‑oriented accounts.

Eligibility criteria were:

- Age ≥18 years;
- English speaking;
- Current clinical practice in genetic counseling, primary care, surgical or medical oncology, or plastic surgery in the United States;
- Direct experience caring for TGD patients considering or undergoing top surgery.

We contacted eligible HCPs in batches to facilitate manageable study flow. Guided by interim analysis of informational power, sample heterogeneity, and interview depth, we determined that 20 interviews were sufficient for robust thematic development.^7^

#### **Data Generation**

To develop our interview guide, we conducted a targeted literature review focused on breast cancer risk assessment and genetic counseling practices for TGD populations, spanning oncology, genetics, and gender-affirming surgery. We piloted the guide with two cancer genetic counselors; feedback informed revisions (pilot data not included in analyses).

Our final semi-structured interview guide (see Supplemental Materials III) explored:

- Current practices and workplace contexts in breast cancer risk assessment and genetic counseling,
- Knowledge and attitudes regarding RRM and GAM for TGD patients,
- Experiences and challenges in discussing cancer risk in gender-affirming care contexts,
- Barriers and facilitators to service delivery,
- Strategies for affirming, equitable communication and care with TGD patients.

We conducted all interviews remotely using HIPAA-compliant Zoom software, supporting national reach and reducing participation barriers. A single interviewer (KB) trained in qualitative methods and LGBTQ+ health conducted the sessions. KB and KZ debriefed following each interview. Both maintained reflexive journals throughout, discussing assumptions, emotional responses, rapport, and initial insights into themes or interpretations to support analytic rigor and reflexivity.

Interviews were audio-recorded with participant consent. We provided a $50 Amazon gift card to each participant alongside a resources sheet detailing best practices and key information for TGD-affirming oncology/genetics care.

#### **Data Management and Analysis**

Audio recordings were transcribed verbatim by a professional service and checked by the study team for accuracy and removal of identifying details. We uploaded transcripts to NVivo software (version 14.23.4)^8^ for management and coding.

We followed reflexive thematic analysis as described by Braun and Clarke:^2–5^

1. Familiarization: We reviewed transcripts and engaged in reflexive journaling;
2. Initial coding: KB and KZ coded the data inductively;
3. Codebook development: We iteratively refined our codebook through discussion with KB, KZ, KFM, and IMM;
4. Theme generation: We identified shared and divergent experiences/practices across our data set;
5. Theme refinement: We checked for coherence, internal consistency, and resonance;
6. Report production: We integrated illustrative quotes and context throughout our interpretation.

Consistent with our social constructivist orientation,^1^ we recognized the co-production of meaning and the influence of researcher and participant positionalities – including clinical discipline, organizational culture, lived experience, and power dynamics. Our team met regularly to review and refine themes. Final thematic development and manuscript drafting incorporated input and critical review from all authors.

To minimize re‑identification risk we report only aggregate demographic data and selected quotations that avoid unique case identifiers; multi‑select intersectional responses are presented as aggregate counts for each option.

**References**

1. Boyland DJR. A social constructivist approach to the gathering of empirical data. In: 2019. https://api.semanticscholar.org/CorpusID:245504109

2. Braun V, Clarke V. Is thematic analysis used well in health psychology? A critical review of published research, with recommendations for quality practice and reporting. *Health Psychol Rev*. 2023;17(4):695-718. doi:10.1080/17437199.2022.2161594

3. Braun V, Clarke V. Supporting best practice in reflexive thematic analysis reporting in Palliative Medicine: A review of published research and introduction to the Reflexive  Thematic Analysis Reporting Guidelines (RTARG). *Palliat Med*. 2024;38(6):608-616. doi:10.1177/02692163241234800

4. Braun V, Clarke V. Reflecting on reflexive thematic analysis. *Qual Res Sport Exerc Health*. 2019;11(4):589-597. doi:10.1080/2159676X.2019.1628806

5. Braun V, Clarke V. Using thematic analysis in psychology. *Qual Res Psychol*. 2006;3(2):77-101. doi:10.1191/1478088706qp063oa

6. Zayhowski K, Blumen K, Mittendorf KF, et al. Transgender and Gender-Diverse Patient Perspectives on Hereditary Breast Cancer Risk Assessment in Gender-Affirming Top Surgeries. *JCO Precis Oncol*. 2025;(9):e2500037. doi:10.1200/PO-25-00037

7. Malterud K, Siersma VD, Guassora AD. Sample Size in Qualitative Interview Studies: Guided by Information Power. *Qual Health Res*. 2016;26(13):1753-1760. doi:10.1177/1049732315617444

8. Lumivero. (2025). NVivo (Version 14.23.4) [Computer software].<https://lumivero.com/products/nvivo/>
