# Supplementary material for: Healthcare professional perspectives on hereditary breast cancer risk assessment prior to gender-affirming mastectomy
Source: Breast Cancer Res Treat. 2026 Jul 29;218(2):22. doi: 10.1007/s10549-026-08038-9 (PMC13421360; doi:10.1007/s10549-026-08038-9)
Supplement: Supplementary file 2 — Supplementary Material 2 [file 10549_2026_8038_MOESM2_ESM.docx]

**SUPPLEMENTAL MATERIALS II: PROVIDER SCREENING/DEMOGRAPHICS SURVEY**

**Purpose of the research study:**

We are interviewing cancer genetic counselors, plastic surgeons, surgical oncologists, and primary care providers who are involved in transgender, non-binary, and gender expansive healthcare and cancer care. We're eager to listen to your perspectives, aiming to enhance the way we evaluate chest and breast cancer risks throughout the top surgery (i.e. chest contouring) experience. We hope that your insights will help shape improved strategies for offering both cancer prevention and gender-affirming care.

**What you will be asked to do in the study:**

You will be asked to complete a voluntary, short survey to see if you are eligible for this study. If you are eligible, you will be asked some demographic questions and then have the choice to enter your email address to be contacted for a virtual interview.

You will then be asked to complete an audio recorded semi-structured interview. The interview will delve into various key areas, including your existing practices within your role, whether it involves caring for transgender, non-binary, and gender expansive patients or specializing in breast cancer care. We'll also discuss the nuances of breast cancer risk assessment and your perspectives on the integration of genetic risk evaluation into the care pathway for individuals contemplating top surgery.

**Time required:**

We anticipate that this survey will take approximately 5 minutes to complete, and that interviews will take approximately 45 to 60 minutes to complete.

**Incentive:**

After completing the virtual interview, you can choose to receive a $50 Amazon gift card through email. Only individuals who complete the virtual interview will be eligible to receive a gift card.

**Risks:**

There are no anticipated risks in this study.

**Benefits:**

There are no direct benefits to you by participating in this study. The study may help to develop new training recommendations for healthcare providers and genetic counselors, suggestions on how to improve patient/provider resources, and direct future research.

**Voluntary participation:**

Participation in the study is voluntary. You have the right to stop the survey or interview at any time. As no identifiable data is linked to the interview responses, you may not withdraw your answers after the entire interview has been completed.

**Confidentiality:**

You will be asked basic demographic information, however you will not be asked to provide any personal identifying information in the recorded interview. We will store your information in ways we think are secure. We will store electronic files in computer systems with password protection and encryption. However, we cannot guarantee complete confidentiality.

This study was approved as an exempt study by the Boston University Institutional Review Board, Study #H-44213.

For questions or concerns regarding your rights as a research participant, you may contact the Boston University Medical Center Institutional Review Board at 617-638-7207.

**If you have questions regarding participation in the study, please contact:**

Kim Zayhowski, MS, CGC (she/her)

Genetic Counselor and Assistant Professor

[kzayhows@bu.edu](mailto:kzayhows@bu.edu)

I wish to participate in this voluntary survey to see if I am eligible for this study.

- Yes
- No [end survey]

**Confirmation of Inclusion Criteria**

Please confirm that the following are true:

- I am 18 years of age or older.
- I am a genetic counselor, breast surgeon (oncology or gender-affirming care), or gender-affirming care provider.
- I am able to speak and read in English.
- I live and practice healthcare in the United States.
- I have read this consent form completely. I understand that my participation is voluntary and that I may quit at any time without penalty.
  - I agree.
  - I disagree.

**Demographic Information**

Thank you for completing the eligibility questions. You are eligible for this study. If you would like to continue with the study, please answer the following demographic questions.

As a reminder, your privacy is of utmost importance to us. The answers to demographic questions will be de-identified. We will store your information in ways we think are secure.

What type of healthcare provider are you?

- Cancer genetic counselor
- Plastic surgeon
  - What type of procedures do you perform?
    - Chest contouring (top surgery)
    - Bilateral risk reducing mastectomies
- Surgical oncologist
  - What type of procedures do you perform?
    - Chest contouring (top surgery)
    - Bilateral risk reducing mastectomies
- Primary care provider who does gender-affirming care
- Not listed, please describe.

What kind of care do you provide to transgender patients?

- Open response

How would you describe your experience with breast cancer care?

- Open response

How do you assess familial risk/genetic risk for cancer?

- Open response

Generally, how would you describe your clinical practice site?

- Academic medical/health center (affiliated with medical school/teaching hospital)
- Community practice (not affiliated with hospital/academic/teaching institution)
- Private practice
- Not listed, please describe [write in option]

What state, territory, or district is your practice in (if you are located in multiple locations, where do you primarily work?)?

- - Alabama
  - Alaska
  - American Samoa
  - Arizona
  - Arkansas
  - California
  - Colorado
  - Connecticut
  - Delaware
  - Florida
  - Georgia
  - Guam
  - Hawaii
  - Idaho
  - Illinois
  - Indiana
  - Iowa
  - Kansas
  - Kentucky
  - Louisiana
  - Maine
  - Maryland
  - Massachusetts
  - Michigan
  - Minnesota
  - Mississippi
  - Missouri
  - Montana
  - Nebraska
  - Nevada
  - New Hampshire
  - New Jersey
  - New Mexico
  - New York
  - North Carolina
  - North Dakota
  - Northern Mariana Islands
  - Ohio
  - Oklahoma
  - Oregon
  - Pennsylvania
  - Puerto Rico
  - Rhode Island
  - South Carolina
  - South Dakota
  - Tennessee
  - Texas
  - U.S. Virgin Islands
  - Utah
  - Vermont
  - Virginia
  - Washington
  - Washington DC
  - West Virginia
  - Wisconsin
  - Wyoming
  - Prefer not to answer

For how many years have you been board certified as a clinician? [Select from drop down - have option for being board eligible, and N/A or “other”]

What type of health insurance is most common in your healthcare practice? [Check all that apply]

- Public health insurance (Medicare, Medicaid, etc.)
- Private health insurance (commercial, employer-based, etc.)
- Uninsured
- Unsure
- Prefer not to respond

How would you describe your racial/ethnic identity? Please select all that apply.

- Black, African American, or African *(For example: African American, Ethiopian, Haitian, Jamaican, Nigerian, Somalian, ect.)*
- American Indian or Alaska Native *(For example: Aztec, Blackfeet Tribe, Mayan, Navajo Nation, Native Village of Barrow Inupiat Traditional Government, Nome Eskimo Community, ect.)*
- Asian (*For example: South Asian, Indian, Chinese, Filipino, Japanese, Korean, Vietnamese, ect.*)
- Middle Eastern or North African *(For example: Algerian, Egyptian, Iranian, Lebanese, Moroccan, Syrian, ect.)*
- Native Hawaiian or other Pacific Islander *(For example: Chamorro, Fijan, Marshallese, Native Hawaiian, Tongan, ect.)*
- Hispanic, Latino, or Spanish *(For example: Colombian, Cuban, Dominican, Mexican, Puerto Rican, Salvodorian, ect.)*
- White *(For example: English, European, French, German, Irish, Italian, Polish, ect.)*
- None of these fully describe me, please specify
- Prefer not to answer

What are your pronouns? *Select all that apply.*

- he/him/him
- she/her/hers
- they/them/their
- zie/zim/zier
- xe/xim/xier
- Not listed
- Prefer not to answer

What is your current gender identity? Select all that apply.

- Man
- Woman
- Genderqueer
- Genderfluid
- Non-binary
- Agender
- Pangender
- Two-Spirit
- Hijra
- Questioning
- Not listed, Please Describe: ________
- Prefer not to answer

Do you identify as transgender or of trans experience?

- Yes, I identify as transgender or of the trans experience
- No, I don’t identify as transgender or of trans experience
- Unsure
- Prefer not to answer

**Communication Information**

Thank you for completing the demographics section. Below you'll find information regarding the paid interview portion.

We aim to interview 20 participants across different specialties. Interviews will take place through Zoom (audio only or video, based on preference) and will last about an hour. The interview will delve into various key areas, including your existing practices within your role, whether it involves caring for transgender, non-binary, and gender expansive patients or specializing in breast cancer care. Interviewees will be compensated with a $50 Amazon gift card.

Please note: Expressing interest in being interviewed does not guarantee that you will be selected for an interview. If you are selected for an interview, you will be provided with further information via email and given the option to consent for that portion of the study. You are free to withdraw your consent and end your participation at any time.

Are you interested in being contacted for an interview?

- Yes
- No [end survey]

Please provide your email address so that we can contact you to set up an interview.

- Free text

If selected for an interview, would you like to be compensated for your participation through a $50 Amazon gift card?

- Yes, I would like the compensation
- No, I am opting out of compensation

What is your first name? Content that you share in your interview will not be tied to your name or email address. We collect names just for the purpose of properly addressing you throughout your participation in this study. (If you prefer to keep your name private, you may provide an alias for the purpose of the interview.)

- Free text

How did you hear about this study ?

- Free text

Is there anything else you would like to share with us?

- Free text

We would like to ask your permission to contact you again in the future. This contact would be after your participation in the study has ended. Please initial your choice below:

____Yes   ____No   You may contact me again to let me know about a different research study”

Question: I am not a robot [click box]
