# Supplementary material for: Healthcare professional perspectives on hereditary breast cancer risk assessment prior to gender-affirming mastectomy
Source: Breast Cancer Res Treat. 2026 Jul 29;218(2):22. doi: 10.1007/s10549-026-08038-9 (PMC13421360; doi:10.1007/s10549-026-08038-9)
Supplement: Supplementary file 3 — Supplementary Material 3 [file 10549_2026_8038_MOESM3_ESM.docx]

**SUPPLEMENTAL MATERIALS III: PROVIDER INTERVIEW GUIDES**

**Pre-interview script:**

My name is *(interviewer name)*, I use *(interviewer pronouns)* pronouns. I’m a *(insert role)* at Boston University. *(Statement of positionality).*

I’m interviewing various healthcare providers who are involved in gender-affirming care and cancer care. We're eager to listen to your perspectives, aiming to enhance the way we evaluate chest and breast cancer risks throughout the top surgery (i.e. chest contouring) experience. We hope that your insights will help shape and improve strategies for offering both cancer prevention and gender-affirming care. The interview will delve into various key areas, including your existing practices within your role, whether it involves caring for transgender, non-binary, and gender expansive patients or specializing in breast cancer care. We'll also discuss the nuances of breast cancer risk assessment and your perspectives on the integration of genetic risk evaluation into the care pathway for transgender, non-binary, and gender expansive individuals contemplating top surgery.

To ensure we accurately include your perspective, this interview will be recorded. We will only use the audio recording of this, and only myself and the *(main investigator/research assistant)* will have access to this recording. You have the option to also turn off your camera and/or change your Zoom name to “Participant *(insert participant number)*” before I start the recording if you wish for the interview. Only two members of the study team, one being myself, one being the (*main investigator/research assistant)*, will be able to link whatever demographic information that you share with me, such as name and email, with your interview. The transcript of the interview will be de-identified for your confidentiality.

Your participation in this interview is completely voluntary. You can choose to skip any question. You can opt to discontinue this interview if you’d like, whenever you’d like. If any of the questions don’t make sense or if you’ve got questions for me, please let me know at any time.

You will receive a $50 Amazon gift card for your participation if you so choose. This will be emailed to you. Can you confirm if the email we scheduled through is the best email to send the gift card?

This interview is expected to take about 30 to 60 minutes to complete.

- What questions do you have before we begin?
- Is it okay if I start recording?
- I am going to turn the recording on now.

# **Primary Care Providers**

**Interviews:**

- Can you please share with me your current role and how long you have been at your current institution?
- What made you interested in participating in this study?

**Section 1: Current Practices and Awareness**

- Could you describe your experience and role as a healthcare provider working with transgender, non-binary, and gender expansive patients considering top surgery?
- What can you tell me about the potential breast cancer risk in people after top surgery?
  - How did you become aware of it?
- How do you approach finding out patient language when discussing breast/chest health?
  - For example, some patients use the word chest, some use the word breast.

**Section 2: Breast Cancer Risk Assessment**

- In your practice, do you currently assess breast cancer risk for patients considering top surgery? If yes, could you describe your approach?
- What factors do you consider when evaluating breast cancer risk in these patients?
  - Prompt: Are there any unique considerations for transgender and gender expansive individuals?
- Are there any challenges you've encountered in assessing breast cancer risk for transgender and gender expansive patients? How have you addressed these challenges?
- How do you approach breast cancer risk assessment and screening for transgender patients who have already undergone top surgery? What are the key considerations in this scenario?
  - Prompt: What challenges have you encountered when determining post-top surgery breast screening strategies, and how have you addressed them? How do you communicate the importance of continued breast screening to transgender patients who have had top surgery?
- Do you feel like the average primary care provider has the training or knowledge to best navigate breast cancer screening and risk for transgender patients?
  - Prompt: Where do you think this education could be incorporated?

**Section 3: Incorporating Breast Cancer Risk Evaluation**

- In your opinion, how important is it to incorporate breast cancer risk assessment into the care of transgender patients considering top surgery? Prompt: Why?
- What strategies or tools do you think could be effective in increasing engagement in breast cancer screening and care for this patient population?
  - Prompt: For example, are there any resources that you think would be useful to give patients?
- What specific resources or guidelines do you think would be helpful for healthcare providers to better assess breast cancer risk in transgender patients?
  - Prompt: How do you discuss breast cancer risk with transgender patients considering top surgery?

**Section 4: Genetic Risk Evaluation**

- Broadly speaking, how do you consider genetic risk evaluation for cancer risks in your practice?
- In your practice, do you consider genetic risk evaluation for transgender patients considering top surgery? If yes, could you describe your approach?
  - How do you approach assessment of when to refer a patient to cancer genetics?
- What factors do you consider when evaluating genetic risk for breast cancer in transgender patients?
- What challenges have you encountered when assessing genetic risk for transgender patients? How have you managed these challenges?
- How do you communicate genetic risk information to transgender patients?
  - Prompt: How do you ensure they fully understand the implications?

Thank you for providing valuable insights into your professional experiences and perspectives. I'd like to delve into the intersection of healthcare and surgical decisions regarding top surgery in relation to cancer risks.

When considering top surgery, particularly for individuals with a genetic predisposition or family history of breast cancer, there are important factors to weigh regarding cancer risk and surgical options. For instance, individuals with a BRCA mutation, which significantly elevates the risk of breast cancer, may opt for risk-reducing bilateral mastectomies to minimize their chances of developing cancer. In contrast, top surgery primarily aims at gender affirmation and may not entail the complete removal of breast tissue, potentially leaving residual tissue and maintaining some level of cancer risk.

- Were you aware of the differences in cancer risk between top surgery and bilateral mastectomies?
  - How do you think this information might impact your practice moving forward?
- How do you think genetic evaluations could better be incorporated into practice?
  - Prompt: Who do you think should be involved in this process?
  - Prompt: Where in the process do you envision a genetic counselor being helpful?
    - In your opinion, what role do you see genetic counselors playing in supporting patients undergoing top surgery? How do you envision collaborating with genetic counselors to ensure patients receive comprehensive care that addresses both their gender-affirming needs and their concerns about cancer risk?
- As a healthcare provider, how do you view the intersection of gender-affirming care and cancer risk management, particularly in the context of patients considering top surgery?
  - Are there any specific challenges or considerations you've encountered in addressing both aspects of care simultaneously?
- When discussing top surgery with patients who have family histories of breast cancer, what information or resources do you believe are most important to provide to help them make informed decisions?
  - How might you integrate discussions about cancer risk assessment and management into your consultations with these patients?
- As we explore the potential for genetic counselors to be involved in the top surgery process, do you have any other thoughts on how they can best support patients?
  - How can we ensure that genetic counseling services are accessible and tailored to meet the diverse needs of individuals undergoing top surgery while prioritizing their overall health and well-being?

**Section 5: Patient Communication and Informed Decision-Making**

- Is there anything you have to add about how you discuss breast cancer risk, genetic risk, and post-top surgery breast screening with transgender patients considering or having undergone top surgery?
  - Prompt: What challenges, if any, have you encountered when communicating these risks to transgender patients, and how have you managed them?
- How can healthcare providers effectively engage transgender patients in shared decision-making regarding breast cancer and genetic risk assessment, as well as surgical options and post-top surgery breast screening?

**Cases:**

- Are there any cases from your time practicing that are closely related to what we’ve discussed here that you would like to share with me?

**Wrapping up**

- What additional information would you like to share with me that I have not addressed?
- It's customary in this kind of study to quote  participants in articles and other reports. The quotes are shared to demonstrate the main themes that the study finds but are not presented in a way that they can be traced back to any individual participant. May I have your permission to quote you, without identifying them as coming from you, in any reports or articles I write from the data from this study?
- What questions, if any, do you have for me before we end the interview?

# **Genetic Counselors**

**Interviews:**

- Can you please share with me your current role and how long you have been at your current institution?
- What made you interested in participating in this study?

**Section 1: Current Practices and Awareness**

- Could you describe your experience and role as a healthcare provider working with transgender, non-binary, and gender expansive patients considering top surgery?
  - How often do you think you see transgender patients?
- What can you tell me about the potential breast cancer risk in people after top surgery?
  - How did you become aware of it?
- How do you approach finding out patient language when discussing breast/chest health?
  - For example, some patients use the word chest, some use the word breast.

**Section 2: Breast Cancer Risk Assessment / Genetic Risk Assessment**

- In your practice, do you currently assess breast cancer risk for patients considering top surgery? If yes, could you describe your approach?
- Could you describe your approach for genetic risk evaluation for transgender patients considering top surgery?
- What factors do you consider when evaluating breast cancer risk in these patients?
  - Prompt: Are there any unique considerations for transgender and gender expansive individuals?
- Are there any challenges you've encountered in assessing breast cancer risk or screening for transgender and gender expansive patients? How have you addressed these challenges?
  - For example, any breast cancer risk calculators, or breast cancer screening?
- How do you communicate genetic risk information to transgender patients?
  - Prompt: How do you ensure they fully understand the implications?
- How do (or would?) you approach breast cancer risk assessment and screening for transgender patients who have already undergone top surgery? What are the key considerations in this scenario?
  - Prompt: What challenges have you encountered (or do you think you’d encounter) when determining post-top surgery breast screening strategies, and how have you addressed them? How do you communicate the importance of continued breast screening to transgender patients who have had top surgery?
- Do you feel like the average genetic counselor has the training or knowledge to best navigate breast cancer screening and risk for transgender patients?
  - Prompt: Where do you think this education could be incorporated?

**Section 3: Incorporating Breast Cancer Risk Evaluation**

- In your opinion, how important is it to incorporate breast cancer risk assessment and genetic risk assessment into the care of transgender patients considering top surgery? Prompt: Why?
- What strategies or tools do you think could be effective in increasing engagement in breast cancer screening and care for this patient population?
  - Prompt: For example, are there any resources that you think would be useful to give patients?
- What specific resources or guidelines do you think would be helpful for healthcare providers to better assess breast cancer risk in transgender patients?
  - Prompt: How do you discuss breast cancer risk with transgender patients considering top surgery?

Thank you for providing valuable insights into your professional experiences and perspectives. I'd like to delve into the intersection of healthcare and surgical decisions regarding top surgery in relation to cancer risks.

When considering top surgery, particularly for individuals with a genetic predisposition or family history of breast cancer, there are important factors to weigh regarding cancer risk and surgical options. For instance, individuals with a BRCA mutation, which significantly elevates the risk of breast cancer, may opt for risk-reducing bilateral mastectomies to minimize their chances of developing cancer. In contrast, top surgery primarily aims at gender affirmation and may not entail the complete removal of breast tissue, potentially leaving residual tissue and maintaining some level of cancer risk.

- Were you aware of the differences in cancer risk between top surgery and bilateral mastectomies?
  - How do you think this information might impact your practice moving forward?
- How do you think genetic evaluations could better be incorporated into practice?
  - Prompt: Who do you think should be involved in this process?
  - Prompt: Where in the process do you envision a genetic counselor being helpful?
    - In your opinion, what role do you see genetic counselors playing in supporting patients undergoing top surgery? How do you envision collaborating with other healthcare providers to ensure patients receive comprehensive care that addresses both their gender-affirming needs and their concerns about cancer risk?
  - Prompt: How do you think this could be standardized?
- As a healthcare provider, how do you view the intersection of gender-affirming care and cancer risk management, particularly in the context of patients considering top surgery?
  - Are there any specific challenges or considerations you've encountered in addressing both aspects of care simultaneously?
- When discussing top surgery with patients who have family histories of breast cancer, what information or resources do you believe are most important to provide to help them make informed decisions?
  - How might you integrate discussions about cancer risk assessment and management into your consultations with these patients?
- As we explore the potential for genetic counselors to be involved in the top surgery process, do you have any other thoughts on how we can best support patients?
  - How can we ensure that genetic counseling services are accessible and tailored to meet the diverse needs of individuals undergoing top surgery while prioritizing their overall health and well-being?

**Section 5: Patient Communication and Informed Decision-Making**

- Is there anything you have to add about how you discuss breast cancer risk, genetic risk, and post-top surgery breast screening with transgender patients considering or having undergone top surgery?
  - Prompt: What challenges, if any, have you encountered when communicating these risks to transgender patients, and how have you managed them?
- How can healthcare providers effectively engage transgender patients in shared decision-making regarding breast cancer and genetic risk assessment, as well as surgical options and post-top surgery breast screening?

**Cases:**

- Are there any cases from your time practicing that are closely related to what we’ve discussed here that you would like to share with me?

**Wrapping up**

- What additional information would you like to share with me that I have not addressed?
- It's customary in this kind of study to quote  participants in articles and other reports. The quotes are shared to demonstrate the main themes that the study finds but are not presented in a way that they can be traced back to any individual participant. May I have your permission to quote you, without identifying them as coming from you, in any reports or articles I write from the data from this study?
- What questions, if any, do you have for me before we end the interview?

# **Oncologists**

**Interviews:**

- Can you please share with me your current role and how long you have been at your current institution?
- What made you interested in participating in this study?

**Section 1: Current Practices and Awareness**

- Could you describe your experience and role as a healthcare provider working with transgender, non-binary, and gender expansive patients considering top surgery or mastectomy?
- What can you tell me about the potential breast cancer risk in people after a cosmetic top surgery?
  - How did you become aware of it?
- How do you approach finding out patient language when discussing breast/chest health?
  - For example, some patients use the word chest, some use the word breast.

**Section 2: Breast Cancer Risk Assessment**

- In your practice, do you currently assess breast cancer risk for patients considering top surgery? If yes, could you describe your approach?
- What factors do you consider when evaluating breast cancer risk in these patients?
  - Prompt: Are there any unique considerations for transgender and gender expansive individuals?
- Are there any challenges you've encountered in assessing breast cancer risk for transgender and gender expansive patients? How have you addressed these challenges?
- Do you feel like the average oncologist has the training or knowledge to best navigate breast cancer screening and risk for transgender patients?
  - Prompt: Where do you think this education could be incorporated?
- Do you educate on the differences between top surgery and mastectomy with transgender patients? Can you tell me more about those conversations?
- How do you guide patients in choosing the most appropriate surgical procedure based on their individual needs, including considerations of gender affirmation and cancer risk?

**Section 3: Incorporating Breast Cancer Risk Evaluation**

- In your opinion, how important is it to incorporate breast cancer risk assessment into the care of transgender patients considering top surgery? Prompt: Why?
- What strategies or tools do you think could be effective in increasing engagement in breast cancer screening and care for this patient population?
  - Prompt: For example, are there any resources that you think would be useful to give patients?
- What specific resources or guidelines do you think would be helpful for healthcare providers to better assess breast cancer risk in transgender patients?
  - Prompt: How do you discuss breast cancer risk with transgender patients considering top surgery?

**Section 4: Genetic Risk Evaluation**

- Broadly speaking, how do you consider genetic risk evaluation for cancer risks in your practice?
- In your practice, do you consider genetic risk evaluation for transgender patients considering top surgery? If yes, could you describe your approach?
  - How do you approach assessment of when to refer a patient to cancer genetics?
- What factors do you consider when evaluating genetic risk for breast cancer in transgender patients?
- What challenges have you encountered when assessing genetic risk for transgender patients? How have you managed these challenges?
- How do you communicate genetic risk information to transgender patients?
  - Prompt: How do you ensure they fully understand the implications?

Thank you for providing valuable insights into your professional experiences and perspectives. I'd like to delve into the intersection of healthcare and surgical decisions regarding top surgery in relation to cancer risks.

When considering top surgery, particularly for individuals with a genetic predisposition or family history of breast cancer, there are important factors to weigh regarding cancer risk and surgical options. For instance, individuals with a BRCA mutation, which significantly elevates the risk of breast cancer, may opt for risk-reducing bilateral mastectomies to minimize their chances of developing cancer. In contrast, top surgery primarily aims at gender affirmation and may not entail the complete removal of breast tissue, potentially leaving residual tissue and maintaining some level of cancer risk.

- Were you aware of the differences in cancer risk between top surgery and bilateral mastectomies?
  - How do you think this information might impact your practice moving forward?
- How do you think genetic evaluations could better be incorporated into practice?
  - Prompt: Who do you think should be involved in this process?
  - Prompt: Where in the process do you envision a genetic counselor being helpful?
    - In your opinion, what role do you see genetic counselors playing in supporting patients undergoing top surgery? How do you envision collaborating with genetic counselors to ensure patients receive comprehensive care that addresses both their gender-affirming needs and their concerns about cancer risk?
- As a healthcare provider, how do you view the intersection of gender-affirming care and cancer risk management, particularly in the context of patients considering top surgery?
  - Are there any specific challenges or considerations you've encountered in addressing both aspects of care simultaneously?
- When discussing top surgery with patients who have family histories of breast cancer, what information or resources do you believe are most important to provide to help them make informed decisions?
  - How might you integrate discussions about cancer risk assessment and management into your consultations with these patients?
- As we explore the potential for genetic counselors to be involved in the top surgery process, do you have any other thoughts on how they can best support patients?
  - How can we ensure that genetic counseling services are accessible and tailored to meet the diverse needs of individuals undergoing top surgery while prioritizing their overall health and well-being?

**Section 5: Patient Communication and Informed Decision-Making**

- Is there anything you have to add about how you discuss breast cancer risk, genetic risk, and post-top surgery breast screening with transgender patients considering or having undergone top surgery?
  - Prompt: What challenges, if any, have you encountered when communicating these risks to transgender patients, and how have you managed them?
- How can healthcare providers effectively engage transgender patients in shared decision-making regarding breast cancer and genetic risk assessment, as well as surgical options and post-top surgery breast screening?

**Cases:**

- Are there any cases from your time practicing that are closely related to what we’ve discussed here that you would like to share with me?

**Wrapping up**

- What additional information would you like to share with me that I have not addressed?
- It's customary in this kind of study to quote  participants in articles and other reports. The quotes are shared to demonstrate the main themes that the study finds but are not presented in a way that they can be traced back to any individual participant. May I have your permission to quote you, without identifying them as coming from you, in any reports or articles I write from the data from this study?
- What questions, if any, do you have for me before we end the interview?

# **Plastic Surgeons**

**Interviews:**

- Can you please share with me your current role and how long you have been at your current institution?
- What made you interested in participating in this study?

**Section 1: Current Practices and Awareness**

- Could you describe your experience and role as a healthcare provider working with transgender, non-binary, and gender expansive patients considering top surgery?
- What can you tell me about the potential breast cancer risk in people after top surgery?
  - How did you become aware of it?
- How do you approach finding out patient language when discussing breast/chest health?
  - For example, some patients use the word chest, some use the word breast.

**Section 2: Breast Cancer Risk Assessment**

- In your practice, do you currently assess breast cancer risk for patients considering top surgery? If yes, could you describe your approach?
- What factors do you consider when evaluating breast cancer risk in these patients?
  - Prompt: Are there any unique considerations for transgender and gender expansive individuals?
- Are there any challenges you've encountered in assessing breast cancer risk for transgender and gender expansive patients? How have you addressed these challenges?
- Do you feel like the average plastic surgeon has the training or knowledge to best navigate breast cancer screening and risk for transgender patients?
  - Prompt: Where do you think this education could be incorporated?
- Do you educate on the differences between top surgery and mastectomy with transgender patients? Can you tell me more about those conversations?
- How do you guide patients in choosing the most appropriate surgical procedure based on their individual needs, including considerations of gender affirmation and cancer risk?

**Section 3: Incorporating Breast Cancer Risk Evaluation**

- In your opinion, how important is it to incorporate breast cancer risk assessment into the care of transgender patients considering top surgery? Prompt: Why?
- What strategies or tools do you think could be effective in increasing engagement in breast cancer screening and care for this patient population?
  - Prompt: For example, are there any resources that you think would be useful to give patients?
- What specific resources or guidelines do you think would be helpful for healthcare providers to better assess breast cancer risk in transgender patients?
  - Prompt: How do you discuss breast cancer risk with transgender patients considering top surgery?

**Section 4: Genetic Risk Evaluation**

- Broadly speaking, how do you consider genetic risk evaluation for cancer risks in your practice?
- In your practice, do you consider genetic risk evaluation for transgender patients considering top surgery? If yes, could you describe your approach?
  - How do you approach assessment of when to refer a patient to cancer genetics?
- What factors do you consider when evaluating genetic risk for breast cancer in transgender patients?
- What challenges have you encountered when assessing genetic risk for transgender patients? How have you managed these challenges?
- How do you communicate genetic risk information to transgender patients?
  - Prompt: How do you ensure they fully understand the implications?

Thank you for providing valuable insights into your professional experiences and perspectives. I'd like to delve into the intersection of healthcare and surgical decisions regarding top surgery in relation to cancer risks.

When considering top surgery, particularly for individuals with a genetic predisposition or family history of breast cancer, there are important factors to weigh regarding cancer risk and surgical options. For instance, individuals with a BRCA mutation, which significantly elevates the risk of breast cancer, may opt for risk-reducing bilateral mastectomies to minimize their chances of developing cancer. In contrast, top surgery primarily aims at gender affirmation and may not entail the complete removal of breast tissue, potentially leaving residual tissue and maintaining some level of cancer risk.

- Were you aware of the differences in cancer risk between top surgery and bilateral mastectomies?
  - How do you think this information might impact your practice moving forward?
- How do you think genetic evaluations could better be incorporated into practice?
  - Prompt: Who do you think should be involved in this process?
  - Prompt: Where in the process do you envision a genetic counselor being helpful?
    - In your opinion, what role do you see genetic counselors playing in supporting patients undergoing top surgery? How do you envision collaborating with genetic counselors to ensure patients receive comprehensive care that addresses both their gender-affirming needs and their concerns about cancer risk?
- As a healthcare provider, how do you view the intersection of gender-affirming care and cancer risk management, particularly in the context of patients considering top surgery?
  - Are there any specific challenges or considerations you've encountered in addressing both aspects of care simultaneously?
- When discussing top surgery with patients who have family histories of breast cancer, what information or resources do you believe are most important to provide to help them make informed decisions?
  - How might you integrate discussions about cancer risk assessment and management into your consultations with these patients?
- As we explore the potential for genetic counselors to be involved in the top surgery process, do you have any other thoughts on how they can best support patients?
  - How can we ensure that genetic counseling services are accessible and tailored to meet the diverse needs of individuals undergoing top surgery while prioritizing their overall health and well-being?

**Section 5: Patient Communication and Informed Decision-Making**

- Is there anything you have to add about how you discuss breast cancer risk, genetic risk, and post-top surgery breast screening with transgender patients considering or having undergone top surgery?
  - Prompt: What challenges, if any, have you encountered when communicating these risks to transgender patients, and how have you managed them?
- How can healthcare providers effectively engage transgender patients in shared decision-making regarding breast cancer and genetic risk assessment, as well as surgical options and post-top surgery breast screening?

**Cases:**

- Are there any cases from your time practicing that are closely related to what we’ve discussed here that you would like to share with me?

**Wrapping up**

- What additional information would you like to share with me that I have not addressed?
- It's customary in this kind of study to quote  participants in articles and other reports. The quotes are shared to demonstrate the main themes that the study finds but are not presented in a way that they can be traced back to any individual participant. May I have your permission to quote you, without identifying them as coming from you, in any reports or articles I write from the data from this study?
- What questions, if any, do you have for me before we end the interview?
